# Supplementary material for: Suppression of trabecular meshwork phagocytosis by norepinephrine is associated with nocturnal increase in intraocular pressure in mice
Source: Commun Biol. 2022 Apr 8;5:339. doi: 10.1038/s42003-022-03295-y (PMC8993819; doi:10.1038/s42003-022-03295-y)
Supplement: Supplementary file 2 — Supplementary information [file 42003_2022_3295_MOESM2_ESM.pdf]

Suppression of trabecular meshwork phagocytosis by norepinephrine is associated with nocturnal increase in intraocular pressure in mice

Authors

Keisuke Ikegami[1*], and Satoru Masubuchi[1]

Affiliations

[1] Department of Physiology, School of Medicine, Aichi Medical University, 1-1 Yazako-karimata, Nagakute, Aichi, 480-1195, Japan

*Correspondence: Keisuke Ikegami, Ph.D.

Department of Physiology, School of Medicine

Aichi Medical University, 1-1 Yazako-karimata, Nagakute 480-1195 Japan

Phone: +81 561 62 3311

Fax: +81 561 63 1289

Email: ikegami.keisuke.910@mail.aichi-med-u.ac.jp

Supplementary information

Table of Contents

1. Supplementary Figure 1
2. Supplementary Figure 2
3. Supplementary Figure 3
4. Supplementary Figure 4
5. Supplementary Figure 5
6. Supplementary Figure 6
7. Supplementary Figure 7
8. Supplementary Figure 8
9. Supplementary Figure 9
10. Supplementary Table 1
11. Supplementary Table 2
12. Material and methods
13. References

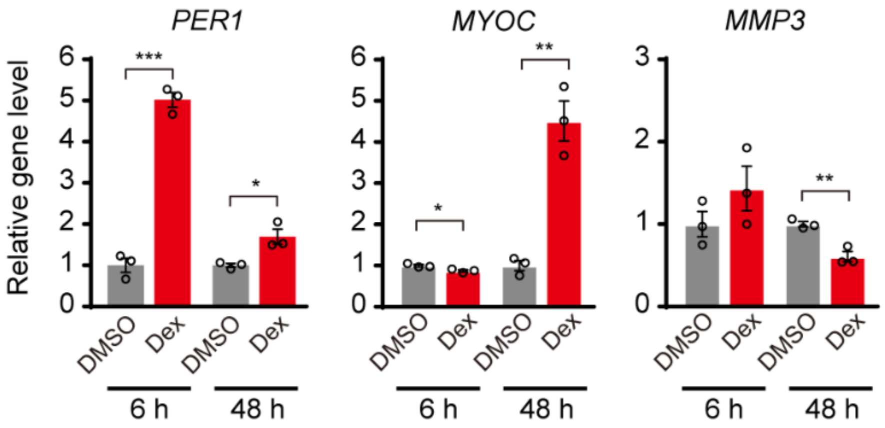

Supplementary Figure 1: Validation of iHTMC by gene expressions analysis. Following dexamethasone (Dex; 100nM) exposure for 6 h and 48 h, the relative gene expression of *PER1*, *MYOC*, and *MMP3* are presented as bar graphs (mean ± SEM) with scatter dot plots of the independent experiments (n = 3). We detected early induction of *PER1*, late induction of *MYOC*, and late suppression of *MMP3* (*p < 0.05, **p < 0.01, ***p < 0.001, one-way ANOVA [p < 0.001], Tukey's multiple comparison).

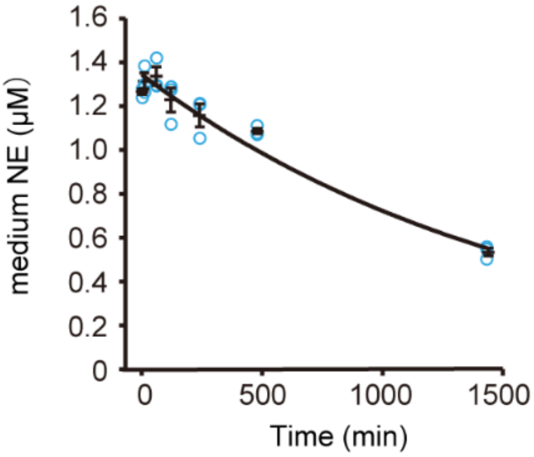

Supplementary Figure 2: Half-life of medium NE *in vitro*.

Temporal changes in noradrenaline (NE) concentration in iHTMC-cultured medium are presented as scatter dot plots with mean ± SEM (n = 3 independent experiments). The half-life of medium NE was 18.6 h.

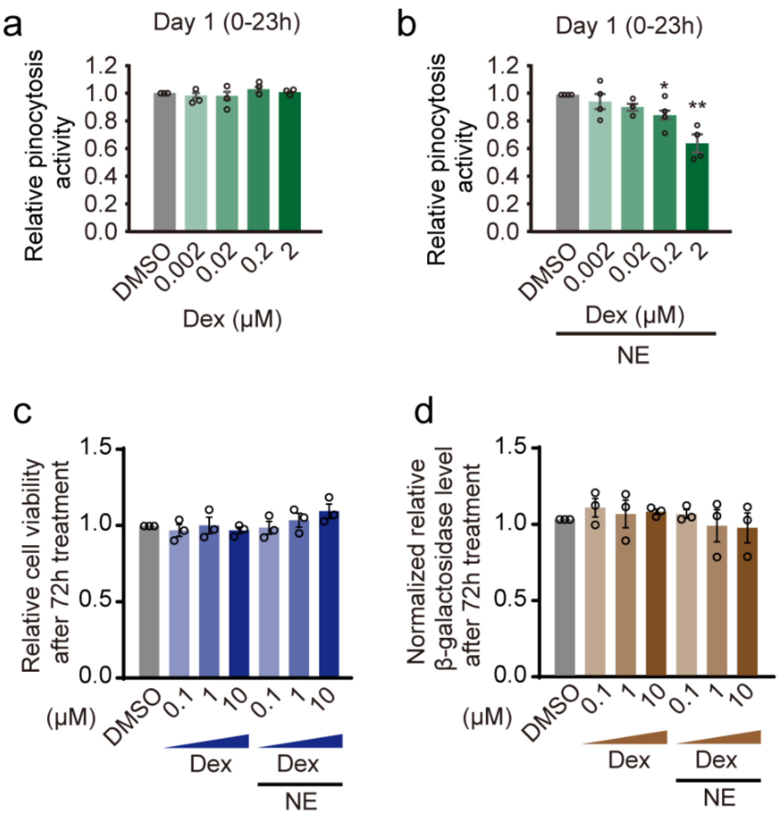

Supplementary Figure 3: Effect of glucocorticoid with NE on the phagocytosis activity, cell viability, and senescence of iHTMC.

a Effect of glucocorticoid analog dexamethasone (Dex) treatment alone on phagocytosis in iHTMC over day 1. Dex alone showed no effect (one-way ANOVA [$P > 0.05$]). **b** Effect of Dex with NE (5 μM) on phagocytosis in iHTMCs during day 1. Dex administration dose-dependently suppressed phagocytosis (*$p < 0.05$, **$p < 0.01$ vs. NE+DMSO, one-way ANOVA [$p < 0.001$], Dunnett's multiple comparison). Phagocytosis activity was normalized to that in the control. **c** Effect of Dex with NE on cell viability in iHTMC after 72 h treatment. Dex with NE showed no change in cell numbers normalized by control DMSO (one-way ANOVA [$p > 0.05$], Dunnett's multiple comparison). **d** Effect of Dex with NE on cell senescence in iHTMCs after 72 h of treatment. Dex with NE showed no effect on the senescence marker β-galactosidase level normalized by cell numbers (one-way ANOVA [$p > 0.05$], Dunnett's multiple comparison). Data (**a-d**) are presented as bar graphs (mean ± SEM) with scatter dot plots of independent experiments (n = 3).

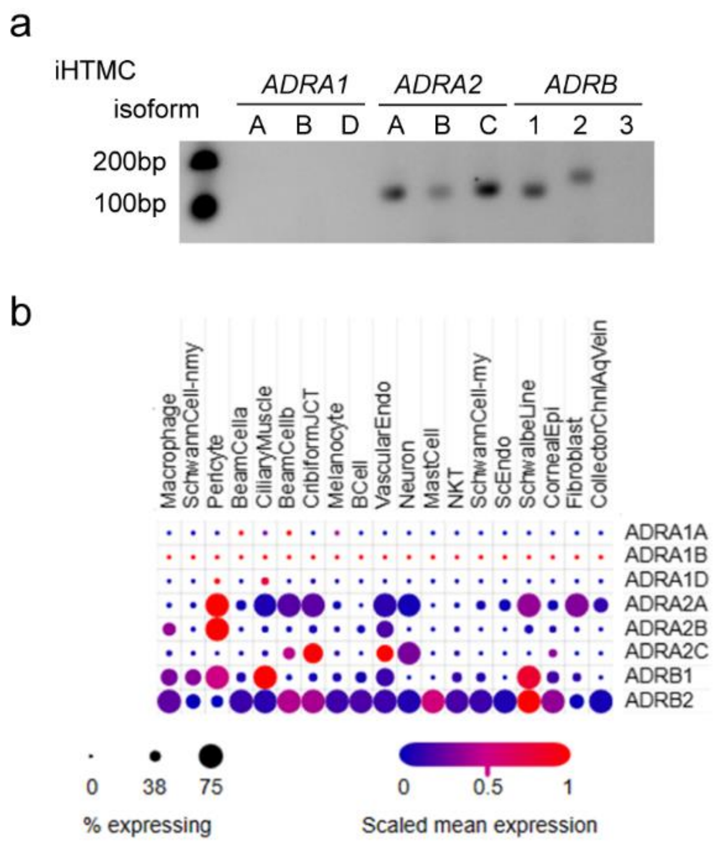

Supplementary Figure 4: Confirmation of AR gene expressions in human AH outflow related cells and iHTMC.

a Reverse transcription-PCR (RT-PCR) revealed strong expression of *ADRA2A*, *ADRA2B*, *ADRA2C*, *ADRB1*, and *ADRB2*, and very weak expression of *ADRA1s* and *ADRB3* in untreated iHTMCs. **b** The *ADRA2B*, *ADRB1*, and *ADRB2* genes are expressed in the TM microphage, while very weak expression of *ADRA1A, ADRA1B, ADRA1D, ADRA2A*, and *ADRA2C* were detected. *ADRA2A* and *ADRA2C* were expressed in other TM cell types, whereas *ADRA2B* levels were decreased. TM cell types: BeamCella, BeamCellb, and CribiformJCT; macrophages: TM macrophages; corneal epithelium: CornealEpi; myelinating Schwann cell: SchwannCell-my; nonmyelinating Schwann cell: SchwannCell-nmy; vascular endothelium: VascularEndo; Schlemm canal endothelium: ScEndo.

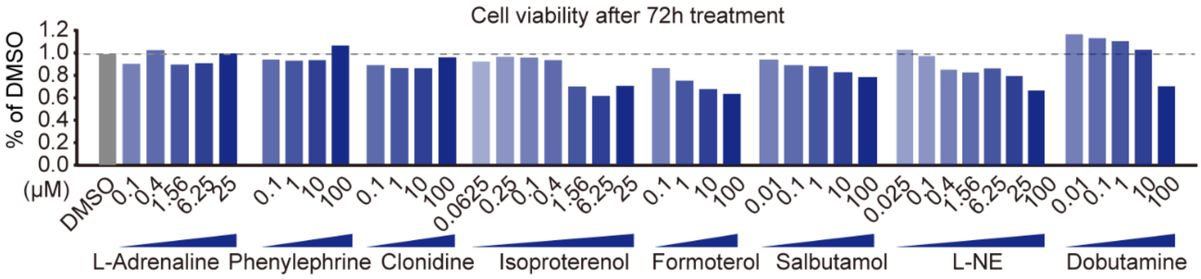

Supplementary Figure 5: Viability of iHTMC by drugs

After 72 h of phagocytosis measurement, iHTMC viability was measured using the cell counting kit-8 assay and normalized to DMSO controls. In particular, isoproterenol and formoterol reduced cell viability in a dose-dependent manner. Based on this result, phagocytosis assays were performed using concentration ranges with less effect on viability. Data are presented as the mean (n = 1; quadruple experiment).

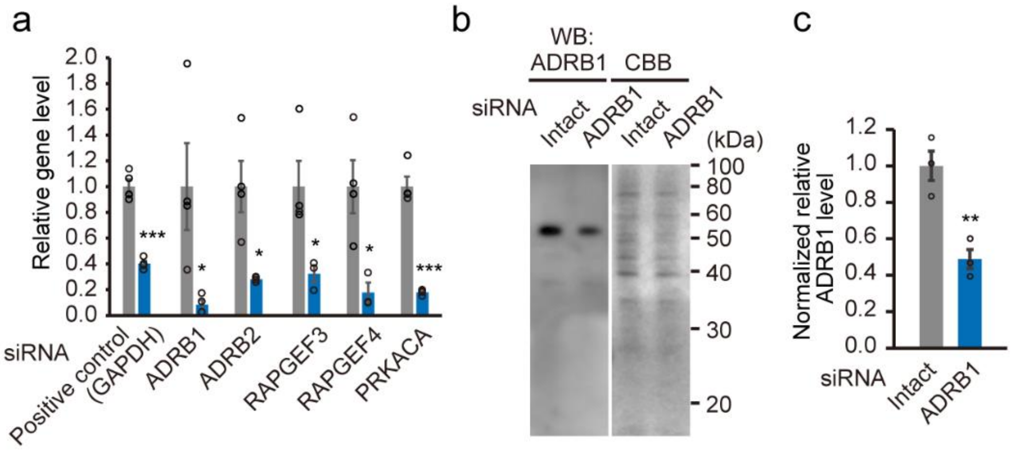

Supplementary Figure 6: Inhibitory effects of preincubation with siRNA on genes and protein level in iHTMC

a Gene expression (positive control *GAPDH*, *ADRB1*, *ADRB2*, *RAPGEF3*, *RAPGEF4*, and *PRKACA*) after 24 h of siRNA exposure presented as scatter dot plots with mean ± SEM (n = 4 independent experiments). All gene levels were significantly suppressed (t-test, *$p < 0.05$, ***$p < 0.001$). **b,c** Western blot analysis revealed that ADRB1 protein levels after siRNA exposure for 24 h were significantly reduced to 48% of the control (no siRNA; Intact) (t-test, **$p < 0.01$). Total proteins for normalization were calculated by CBB staining using the same membrane. **c** Data are normalized by total protein and presented as bar graphs (mean ± SEM) with scatter dot plots of independent experiments (n = 3).

Fig. 5b Western blots used for densitometry quantification

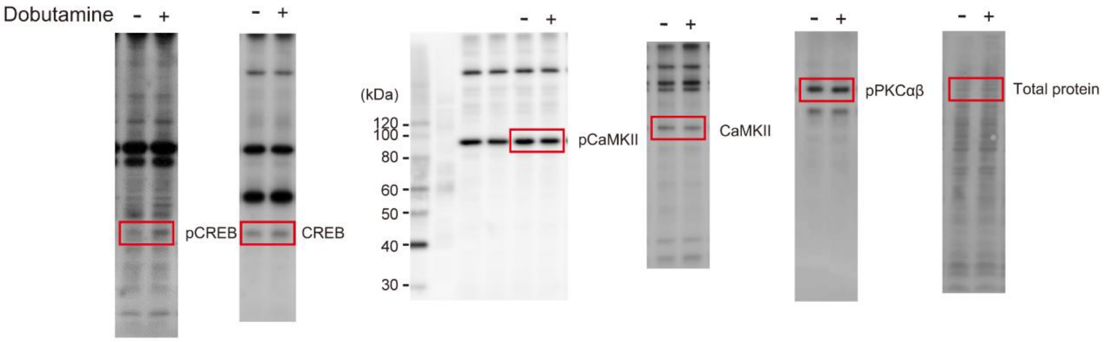

Fig. 6d Western blots used for densitometry quantification

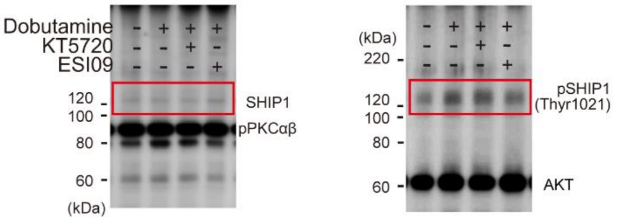

Fig. 6e Western blots used for densitometry quantification

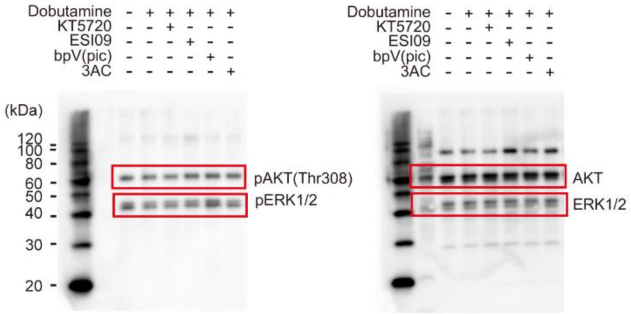

Supplementary Figure 7: Images for uncropped blots

Uncropped images of western blots from the corresponding cropped western blots, shown in the main text. Molecular weight markers are shown. Figure subpanel is indicated for each blot. CREB, cyclic adenosine monophosphate response element binding protein

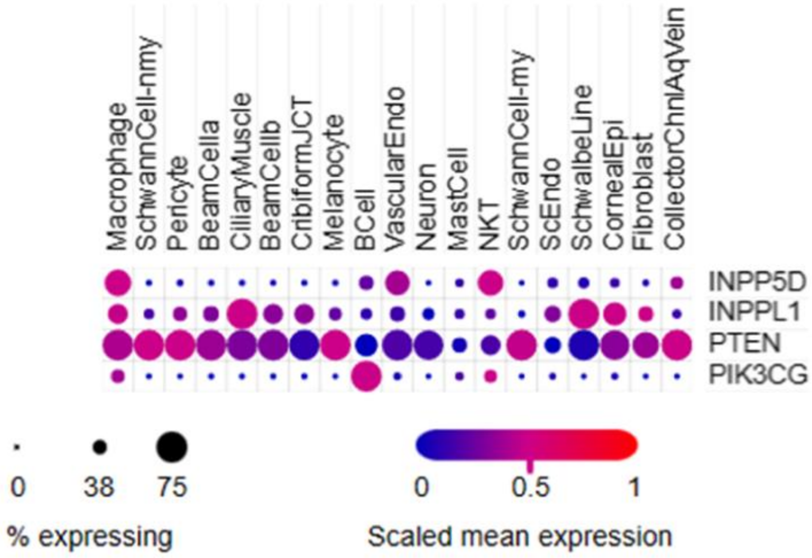

Supplementary Figure 8: Gene expressions of SHIP1, SHIP2, PTEN, and PIK3γ in human AH outflow-related cells

SHIP1 (*INPP5D*), SHIP2 (*INPPL1*), PTEN, and PIK3γ (*PIK3CG*) were expressed in TM macrophages. In particular, *INPP5D* and *PTEN* levels were higher than those of *INPPL1* and *PIK3CG*. TM cell types: BeamCella, BeamCellb, and CribiformJCT; macrophages: TM macrophages; corneal epithelium: CornealEpi; myelinating Schwann cell: SchwannCell-my; nonmyelinating Schwann cell: SchwannCell-nmy; vascular endothelium: VascularEndo; Schlemm canal endothelium: ScEndo.

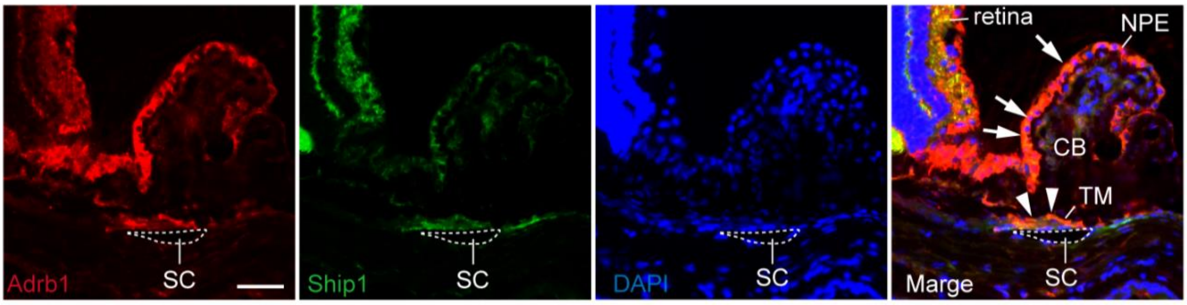

Supplementary Figure 9: Localization of Adrb1 and Ship1 in the mouse eye

Immunohistochemistry revealed co-localization of Adrb1 (red) and Ship1 (green) in the endothelial cells of the Schlemm's canal (arrowhead) and non-pigmented epithelial cells in the ciliary body (arrow) of the B6 mouse eye. SC, Schlemm's canal; CB, ciliary body; NPE, non-pigmented epithelial cell; TM, trabecular meshwork. Scale bar = 100 µm.

Catalog Number	Gene Symbol	Gene Accession	Sequence
D-001930-01	Accell GAPD Control		GUGUGAACCAUGAGAAGUA
D-001930-12	Accell GAPD Control		UUUACAUGUUCCAAUAUGA
D-001930-13	Accell GAPD Control		CUGCCAAAUAUGAUGACAU
D-001930-14	Accell GAPD Control		CUGGUAUGACAACGAAUUU
D-001910-01	Accell Non-targeting Control		UGGUUUACAUGUCGACUAA
D-001910-02	Accell Non-targeting Control		UGGUUUACAUGUUUUCUGA
D-001910-03	Accell Non-targeting Control		UGGUUUACAUGUUUUCCUA
D-001910-04	Accell Non-targeting Control		UGGUUUACAUGUUGUGUGA
A-005425-14	*ADRB1*	NM_000684	GUAUCAAUAUUAGUUGGAA
A-005425-15	*ADRB1*	NM_000684	CUGUUGUCUAGUAUGUUAU
A-005425-16	*ADRB1*	NM_000684	UGAUGUUCCUUGUUGUUUU
A-005425-17	*ADRB1*	NM_000684	GGGCAGAUCUUAAAUAAAA
A-005426-14	*ADRB2*	NM_000024	GGAGCAGCUUCAGUUGUUU
A-005426-15	*ADRB2*	NM_000024	CUAUUUAACUUGAGGGUAA
A-005426-16	*ADRB2*	NM_000024	UGAUUAUUUGUUAUUUGUA
A-005426-17	*ADRB2*	NM_000024	CAUGGAAUUUGUAAGUUUA
A-007676-13	*RAPGEF3*	NM_006105	CGGUGAAGCGAGAAUUAGC
A-007676-14	*RAPGEF3*	NM_006105	UGGUGAAGGUCAAUUCUGC
A-007676-15	*RAPGEF3*	NM_006105	GGAUCUGUCAACGUGGUGA
A-007676-16	*RAPGEF3*	NM_006105	CGAGGAUUUCCACAUGCUC
A-009511-13	*RAPGEF4*	NM_007023	UGCUCAAACCUAAUGAUGU
A-009511-14	*RAPGEF4*	NM_007023	UCGUCGACCAUAGACAUUU
A-009511-15	*RAPGEF4*	NM_007023	UUCUGGACUUAAAUGUUAU
A-009511-16	*RAPGEF4*	NM_007023	UUGACAUGCCUAAAGCUUA
A-004649-13	*PRKACA*	NM_207518	CGAGUAACUUUGACGACUA
A-004649-14	*PRKACA*	NM_207518	GUGACAUGUUCAGUGGGUU
A-004649-15	*PRKACA*	NM_207518	CCUUCAUACCAAAGUUUAA
A-004649-16	*PRKACA*	NM_207518	GUCCUGACCUUUGAGUAUC

Supplementary Table 1: Accell SMARTpool siRNA data, related to Fig. 4, Fig. 5, and Supplementary Figure 6.

Gene	Forward (5'→3')	Reverse (5'→3')
PER1	TCAACTGCCTGGACAGCATCCT	TCAGAGGCTGAGGAGGTGGTAT
MYOC	CCATCTGGCTATCTCAGGAGTG	GCATCCACACACCATACTTGCC
MMP3	CACTCACAGACCTGACTCGGTT	AAGCAGGATCACAGTTGGCTGG
ADRA1D	GAAGAAAGCGGCCAAGACTCTG	GAAGTAGCCGAGCCAGAAGATG
ADRA1B	GCAGCTAAGACGTTGGGCATTG	TTCAGGGTGGAGAACAAGGAGC
ADRA1A	TCTCCAAGACGGATGGCGTTTG	TGGTGACAGAGCGAGACTTCGT
ADRA2A	CTTCTGGTTCGGCTACTGCAAC	GGAAACCTCACACGATCCGCTT
ADRA2B	TCTTCGGCAACGCTCTGGTCAT	CCAGCGAGAAAGGGATGATGAG
ADRA2C	AGCTACAGCCTGTACGGCATCT	CGGAAATCCTGGTTGAAGACCG
ADRB1	TTCCTGCCCATCCTCATGCACT	GTAGAAGGAGACTACGGACGAG
ADRB2	TACCAGAGCCTGCTGACCAAGA	AGTCACAGCAGGTCTCATTGGC
ADRB3	AGCCCAGGCTTTGCCAACGGC	GGGACTCATTCTGAACAGAGGC
RAPGEF3	GTCATTTCCTGCGTGTGGACAAG	CCACTTTGCCATGTTCTTCCAGC
RAPGEF4	GTATGGAGACCTCCTGCAAGAG	CAACTCTGGCAGTTGCTCCTTG
PRKACA	CCACTATGCCATGAAGATCCTCG	CGAGTTTGACGAGGAACGGAAAG
GAPDH	GTCTCCTCTGACTTCAACAGCG	ACCACCCTGTTGCTGTAGCCAA
RPL13A	CTCAAGGTGTTTGACGGCATCC	TACTTCCAGCCAACCTCGTGAG

Supplementary Table 2 Primer sequences used in RT-PCR and qPCR, related to Supplementary Figures 1, 4, and 6.

Material and Methods

Half-life of medium noradrenaline

To calculate the half-life of noradrenaline (NE) in the medium, we collected the medium including initially 1 µM NE, 0, 10, 60, 120, 240, 480, and 1440 min after adding to iHTMC culture. NE was measured using Noradrenaline Research ELISA (E-5200R, LDN, Nordhorn, Germany), according to the manufacturer's instructions. Briefly, 50 µL of the medium was used for the extraction and acylation. After extraction with 25 mM HCl, 90 µL of the supernatant was used for the subsequent enzymatic conversion using catechol-O-methyltransferase and S-adenosyl-L-methionin. After 4-fold dilution, 100µL of the supernatant were transferred to an antigen pre-coated Noradrenaline microplate and incubated with NE antiserum overnight at 4 °C. After incubation with peroxidase-labeled goat anti-rabbit IgG, the color-developing substrate tetramethylbenzidine (TMB) was added. After color development by incubating at 37 °C for 30 min, the absorbance at 450 nm and 620 nm (reference) was measured using a SpectraMax M5 (Molecular Devices Japan, Tokyo, Japan). After correction with 620 nm absorbance, the NE concentration was fitted to the equation $y = a*e^{-kt}$, where y is the NE concentration and t is time. The half-life was calculated as $t_{1/2} = \ln(2) / k$.

Viability Assays

After 72 h of fluorescent measurement, 10 µL of cell counting kit-8 (CK04, Dojindo) was added to cells seeded in quadruplicate in 96 wells, and incubated for 4 h in a 37 °C CO_2 incubator. To calculate cell numbers, iHTMC were preincubated in collagen I coated 96-well microplates (4860-010; IWAKI) at densities of 16 × 10^3, 8.0 × 10^3 , 4.0 × 10^3, 2.0 × 10^3, 1.0 × 10^3, and 0.5 × 10^3 cells/well with serum free TMCM (1% penicillin/streptomycin;

#6591; Sciencell), and were counted as standard. Subsequently, the absorbance (450 nm) was measured using a SpectraMax M5 (Molecular Device).

β-galactosidase assays

To measure SA-β-galactosidase (senescent cell marker), we used the Cellular Senescence Detection Kit -SPiDER-βGal protocol (SG02, DOJINDO), following the manufacturer's instructions. Briefly, after cell counting, iHTMCs were washed once with PBS and lysed with lysis buffer to extract cellular SA-β-galactosidase. The lysate solution was added to the same volume of SPiDER-βGal working solution and incubated at 37 °C for 30 min. After adding the stop solution, fluorescence (Ex = 500 nm; Em = 540 nm) was measured using a SpectraMax M5 (Molecular Device).

Analysis of gene expression using Public Data

We performed data analysis of gene expression in human AH outflow-related cells from the Gene Expression Omnibus accession number GSE146188 [1]. Data can be visualized in the Broad Institute's Single Cell Portal at https://singlecell.broadinstitute.org/single_cell/study/SCP780.

Reverse Transcription-PCR (RT-PCR)

We isolated total RNA from iHTMC using NucleoSpin RNA/Protein (U0933A, Takara Bio Inc., Japan), according to the manufacturer's instructions. After genomic DNA removal by DNase I treatment (Toyobo, Osaka, Japan), a 0.5 µg aliquot of each total RNA preparation was reverse transcribed using ReverTra Ace qPCR RT Master Mix with gDNA Remover (Toyobo, Osaka, Japan) and 2.5 µM oligo (dT) as previously described

[2]. cDNA was subjected to PCR using a buffer containing 0.2 U/10 μL Taq DNA polymerase, 45 mM KCl, 2.5 mM Mg^{2+}, 200 μM dNTP (Blend Taq Plus, TB-BTQ-201, Toyobo) and 0.4 μM of the corresponding primers. The PCR conditions were 95 °C for 2 min, followed by 35 cycles at 94 °C for 15 s, 60 °C for 15 s, and 72 °C for 45 s, with a final extension at 72 °C for 5 min. PCR products were resolved on a 2 % agarose gel in Tris-acetate-EDTA buffer (TAE). RT-PCR for the expression of nine genes (*ADRA1A, ADRA1B, ADRA1D, ADRA2A*, *ADRA2B*, *ADRA2C*, *ADRB1*, *ADRB2*, and *ADRB3*) was performed with each of the two primers (Origene), as described in Supplementary Table 2.

Quantitative PCR

Real-time PCR (qPCR) was performed as previously described [2] using an ABI StepOne plus, in a total volume of 10 μL using KOD SYBR qPCR Mix (TB-QKD-201, Toyobo), according to the supplier's instructions. mRNA quantification was performed using two primers (Origene), as described in Supplementary Table 2. The resulting threshold cycle (Ct) values from the cDNA amplifications were normalized to the Ct values for *GAPDH* or *RPL13A*.

Immunohistochemistry

Paraffin sections of paraformaldehyde-fixed C57BL/6J eyes (5 μm) were used for immunofluorescence [3]. After complete removal of paraffin, the sections were immersed in HistoVT One (pH7.0, # 06380, Nakarai) and heated in a microwave oven for 15 min at low voltage. After blocking with normal horse serum (Vectastain), sections were incubated with rabbit polyclonal antibody against INPP5D (SHIP1) (1:200; A0122,

ABclonal), and goat polyclonal antibody against ADRB1 (1:500; NB600-978, Novus Biologicals) at 4 °C for 48 h. This was followed by incubation with donkey polyclonal Alexa Fluor 488-conjugated anti-rabbit IgG (1:400; R37118, Thermo Fisher) and donkey polyclonal DyLight 549-conjugated anti-gpat IgG (1:400) secondary antibody for 120 min (1:400; R37118, Thermo Fisher), including DAPI (1:1000; LH166, Dojindo), as a counter stain. After encapsulation with mounting medium, fluorescent images were detected using a BZ-X800 (Keyence).

References

1. van Zyl, T. *et al.* Cell atlas of aqueous humor outflow pathways in eyes of humans and four model species provides insight into glaucoma pathogenesis. *Proceedings of the National Academy of Sciences of the United States of America* **117**, 10339-10349 (2020).

2. Ikegami, K. *et al.* Effect of expression alteration in flanking genes on phenotypes of St8sia2-deficient mice. *Scientific Reports* **9**, 1-11 (2019).

3. Ikegami, K., Shigeyoshi, Y. & Masubuchi, S. Circadian regulation of IOP rhythm by dual pathways of glucocorticoids and sympathetic nervous system. *Invest Ophthalmol Visual Science* **61**, 26 (2020).
