# Supplementary material for: Suppression of trabecular meshwork phagocytosis by norepinephrine is associated with nocturnal increase in intraocular pressure in mice
Source: Commun Biol. 2022 Apr 8;5:339. doi: 10.1038/s42003-022-03295-y (PMC8993819; doi:10.1038/s42003-022-03295-y)
Supplement: Supplementary file 15 — Reporting Summary [file 42003_2022_3295_MOESM15_ESM.pdf]

nature portfolio

Corresponding author(s): Keisuke Ikegami

Last updated by author(s): Feb 25, 2022

Reporting Summary

Nature Portfolio wishes to improve the reproducibility of the work that we publish. This form provides structure for consistency and transparency in reporting. For further information on Nature Portfolio policies, see our Editorial Policies and the Editorial Policy Checklist.

Statistics

For all statistical analyses, confirm that the following items are present in the figure legend, table legend, main text, or Methods section.

n/a	Confirmed	
☐	☒	The exact sample size (n) for each experimental group/condition, given as a discrete number and unit of measurement
☐	☒	A statement on whether measurements were taken from distinct samples or whether the same sample was measured repeatedly
☐	☒	The statistical test(s) used AND whether they are one- or two-sided<br>*Only common tests should be described solely by name; describe more complex techniques in the Methods section.*
☒	☐	A description of all covariates tested
☒	☐	A description of any assumptions or corrections, such as tests of normality and adjustment for multiple comparisons
☐	☒	A full description of the statistical parameters including central tendency (e.g. means) or other basic estimates (e.g. regression coefficient) AND variation (e.g. standard deviation) or associated estimates of uncertainty (e.g. confidence intervals)
☐	☒	For null hypothesis testing, the test statistic (e.g. F, t, r) with confidence intervals, effect sizes, degrees of freedom and P value noted<br>*Give P values as exact values whenever suitable.*
☒	☐	For Bayesian analysis, information on the choice of priors and Markov chain Monte Carlo settings
☒	☐	For hierarchical and complex designs, identification of the appropriate level for tests and full reporting of outcomes
☒	☐	Estimates of effect sizes (e.g. Cohen's d, Pearson's r), indicating how they were calculated

Our web collection on statistics for biologists contains articles on many of the points above.

Software and code

Policy information about availability of computer code

Data collection	IncuCyte ZOOM (Essen Bioscience)<br>Tonometer (Icare TonoLab, TV02)<br>Digital fluorescent microscope Dino-Lite Edge M Fluorescence TGFBW (Opto Science Inc.)<br>SpectraMax M5 (Molecular Devices)<br>Amersham Imager 600 (Cytiva Lifescience)<br>StepOne plus (ABI)<br>BZ-X800 (Keyence)
Data analysis	IncuCyte ZOOM 2015A software (Essen Bioscience)<br>GraphPad Prism 6.0 software (GraphPad Software Inc.)<br>Excel-Toukei 2012 software (Social Survey Research Information Co. Ltd.)<br>SpectraMax M5 (Molecular Devices)<br>Amersham Imager 600 (Cytiva Lifescience)<br>StepOne plus (ABI)

For manuscripts utilizing custom algorithms or software that are central to the research but not yet described in published literature, software must be made available to editors and reviewers. We strongly encourage code deposition in a community repository (e.g. GitHub). See the Nature Portfolio guidelines for submitting code & software for further information.

Data

Policy information about availability of data

All manuscripts must include a data availability statement. This statement should provide the following information, where applicable:

- Accession codes, unique identifiers, or web links for publicly available datasets
- A description of any restrictions on data availability
- For clinical datasets or third party data, please ensure that the statement adheres to our policy

Data analysis of gene expressions in human AH outflow-related cells (https://singlecell.broadinstitute.org/single_cell/study/SCP780) were analyzed at Broad Institute's Single Cell Portal (GSE146188). Primer data of qPCR and RT-PCR were provided as Supplementary table 1-2. Unprocessed images of all western blots are presented in Supplementary Fig 7. All other datasets generated during in this study are available from the corresponding authors upon reasonable request.

Field-specific reporting

Please select the one below that is the best fit for your research. If you are not sure, read the appropriate sections before making your selection.

☒ Life sciences ☐ Behavioural & social sciences ☐ Ecological, evolutionary & environmental sciences

For a reference copy of the document with all sections, see nature.com/documents/nr-reporting-summary-flat.pdf

Life sciences study design

All studies must disclose on these points even when the disclosure is negative.

Sample size	No statistical methods were used to predetermine sample size. The number of mice used was set to N = 6 or more because individual differences are likely to occur in the IOP data. Sample sizes of at least 3 independent experimental replicates were used based on previous experience and the standard practices of the field.
Data exclusions	No data were excluded from the analysis
Replication	All experiments were performed in at least biological triplicate with similar results. Where only one biological repeat was performed for screening, this is noted in the figure legends. In the animal experiments of intraocular pressure measurement, positive control was included in the independent measurement.
Randomization	Inbread mice of the same age and sex were bred in the same environment and used in the experiment. Intraocular pressure measurements were performed randomly. Randomization was not required for the type of in vitro data we have reported in this study
Blinding	No blinding was used in this study

Reporting for specific materials, systems and methods

We require information from authors about some types of materials, experimental systems and methods used in many studies. Here, indicate whether each material, system or method listed is relevant to your study. If you are not sure if a list item applies to your research, read the appropriate section before selecting a response.

Materials & experimental systems

n/a	Involved in the study	
☐	☒	Antibodies
☐	☒	Eukaryotic cell lines
☒	☐	Palaeontology and archaeology
☐	☒	Animals and other organisms
☒	☐	Human research participants
☒	☐	Clinical data
☒	☐	Dual use research of concern

Methods

n/a	Involved in the study	
☒	☐	ChIP-seq
☒	☐	Flow cytometry
☒	☐	MRI-based neuroimaging

Antibodies

Antibodies used

Rabbit anti- phospho-CREB (Ser133) Cell Signalling Technology Cat#9198
Rabbit anti- CREB Cell Signalling Technology Cat#9192
Rabbit anti- Akt (pan) Cell Signalling Technology Cat#4691
Rabbit anti- phospho-PKCα/βII (Thr638/641) Cell Signalling Technology Cat#9375
Rabbit anti- phospho-Akt (Thr308) Cell Signalling Technology Cat#9275

	Rabbit anti- phosphor-CaMKII alpha (Thr286) Abcam Cat# ab5683<br>Rabbit anti- ERK1 / ERK2 ABclonal Cat# A16686<br>Rabbit anti- phospho-ERK1(T202/Y204) /ERK2(T185/Y187) ABclonal Cat# AP0472<br>Rabbit anti- INPP5D (SHIP1) ABclonal Cat# A0122<br>Rabbit anti- phosphor-INPP5D (Tyr1021) CSB Cat# PA903060<br>Mouse anti- CaMKIIα/β/γ/δ (G-1) Santa Cruz Biotechnology Cat# sc-5306<br>HRP-conjugated goat polyclonal antibody against rabbit IgG Cell Signalling Technology Cat# 7074<br>HRP-conjugated goat polyclonal antibody against mouse IgG Cell Signalling Technology Cat# 7076<br>Goat anti-ADRB1 Novus Biologicals Cat# NB600-978
Validation	All antibodies were validated by commercial source. No homemade or previously unpublished antibodies were used in this study. All antibodies were validated using western blotting and/or immunofluorescence staining.

Eukaryotic cell lines

Policy information about cell lines

Cell line source(s)	Immortalized human trabecular meshwork cell (T0371-C) (Applied Biological Materials Inc.)
Authentication	This iHTMC identity used in this study was confirmed by cell growth speed and phagocytosis array.
Mycoplasma contamination	This cell line was not tested for mycoplasma contamination.
Commonly misidentified lines<br>(See ICLAC register)	No commonly misidentified cell lines were used in this study.

Animals and other organisms

Policy information about studies involving animals; ARRIVE guidelines recommended for reporting animal research

Laboratory animals	Five-week-old male C57BL/6JJmsSlc.
Wild animals	This study did not involve wild animals.
Field-collected samples	This study did not involve samples collected from the field.
Ethics oversight	All animal experiments were approved by the Committee of Animal Care and Use of the Aichi Medical University. All experimental procedures were conducted in accordance with the institutional guidelines for the use of experimental animals.

Note that full information on the approval of the study protocol must also be provided in the manuscript.
